# Interspecies Transmission of CMY-2-Producing Escherichia coli Sequence Type 963 Isolates between Humans and Gulls in Australia

**DOI:** 10.1128/msphere.00238-22

**Published:** 2022-07-05

**Authors:** Matej Medvecky, Costas C. Papagiannitsis, Ethan R. Wyrsch, Ibrahim Bitar, Max L. Cummins, Steven P. Djordjevic, Monika Dolejska

**Affiliations:** a Central European Institute of Technology, University of Veterinary Sciences Brno, Brno, Czech Republic; b Department of Chemistry, Faculty of Science, University of Hradec Kralove, Hradec Kralove, Czech Republic; c Institute of Biodiversity, Animal Health & Comparative Medicine, University of Glasgow, Glasgow, United Kingdom; d Department of Microbiology, University Hospital of Larissa, Larissa, Greece; e Australian Institute for Microbiology and Infection, University of Technology Sydney, Ultimo, Australia; f The Australian Centre for Genomic Epidemiological Microbiology, University of Technology Sydney, Ultimo, Australia; g Faculty of Medicine, Biomedical Center, Charles University, Plzen, Czech Republic; h Department of Biology and Wildlife Diseases, Faculty of Veterinary Hygiene and Ecology, University of Veterinary Sciences Brno, Brno, Czech Republic; i Department of Clinical Microbiology and Immunology, Institute of Laboratory Medicine, The University Hospital Brno, Czech Republic; JMI Laboratories

**Keywords:** β-lactamases, *Escherichia coli*, ST963, WGS, Australia, transmission, comparative genomics, phylogenetic analysis, silver gulls, humans

## Abstract

Escherichia coli sequence type 963 (ST963) is a neglected lineage closely related to ST38, a globally widespread extraintestinal pathogenic ST causing urinary tract infections (UTI) as well as sepsis in humans. Our current study aimed to improve the knowledge of this understudied ST by carrying out a comprehensive comparative analysis of whole-genome sequencing data consisting of 31 isolates from silver gulls in Australia along with another 80 genomes from public resources originating from geographically scattered regions. ST963 was notable for carriage of cephalosporinase gene *bla*_CMY-2,_ which was identified in 99 isolates and was generally chromosomally encoded. ST963 isolates showed otherwise low carriage of antibiotic resistance genes, in contrast with the closely related E. coli ST38. We found considerable phylogenetic variability among international ST963 isolates (up to 11,273 single nucleotide polymorphisms [SNPs]), forming three separate clades. A major clade that often differed by 20 SNPs or less consisted of Australian isolates of both human and animal origin, providing evidence of zoonotic or zooanthropogenic transmission. There was a high prevalence of virulence F29:A-:B10 pUTI89-like plasmids within E. coli ST963 (*n* = 88), carried especially by less variable isolates exhibiting ≤1,154 SNPs. We characterized a novel 115,443-bp pUTI89-like plasmid, pCE2050_A, that carried a *traS*:IS*5* insertion absent from pUTI89. Since IS*5* was also present in a transposition unit bearing *bla*_CMY-2_ on chromosomes of ST963 strains, IS*5* insertion into pUTI89 may enable mobilization of the *bla*_CMY-2_ gene from the chromosome/transposition unit to pUTI89 via homologous recombination.

**IMPORTANCE** We have provided the first comprehensive genomic study of E. coli ST963 by analyzing various genomic and phenotypic data sets of isolates from Australian silver gulls and comparison with genomes from geographically dispersed regions of human and animal origin. Our study suggests the emergence of a specific *bla*_CMY-2_-carrying E. coli ST963 clone in Australia that is widely spread across the continent by humans and birds. Genomic analysis has revealed that ST963 is a globally dispersed lineage with a remarkable set of virulence genes and virulence plasmids described in uropathogenic E. coli. While ST963 separated into three clusters, a unique specific clade of Australian ST963 isolates harboring a chromosomal copy of AmpC β-lactamase encoding the gene *bla*_CMY-2_ and originating from both humans and wild birds was identified. This phylogenetically close cluster comprised isolates of both animal and human origin, thus providing evidence of interspecies zoonotic transmission. The analysis of the genetic environment of the AmpC β-lactamase-encoding gene highlighted ongoing evolutionary events that shape the carriage of this gene in ST963.

## INTRODUCTION

Extended-spectrum β-lactamases (ESBL) and AmpC type β-lactamases are enzymes that are able to hydrolyze beta-lactams including extended-spectrum cephalosporins and are frequently found in *Enterobacterales*, such as Escherichia coli and Klebsiella pneumoniae, of human and animal origin (1). The most common plasmid-mediated AmpC β-lactamase in *Enterobacterales* is CMY-2 (2). The gene encoding CMY-2 (*bla*_CMY-2_) likely originates from a chromosomal AmpC gene from Citrobacter freundii (3) and was translocated onto plasmids by IS*Ecp1* (4). Consequently, the gene has spread to different hosts by conjugative plasmids belonging to various incompatibility (Inc) groups, including A, C2, I1, F, N, K/B, and K2 (5–10).

E. coli sequence type 963 (ST963) is a relatively understudied lineage belonging to clonal complex (CC) 38 and phylogroup D. E. coli ST38 is an extraintestinal pathogenic (ExPEC) bacterium that is a documented cause of urinary tract infections and sepsis in humans (11). Moreover, E. coli ST38 is geographically widespread and is associated with multidrug resistance (MDR) and ESBL production, making it an important pandemic ST globally (11). To our knowledge, there is a lack of genomic studies comprising broader collections of ST963 isolates. *bla*_CMY-2_-carrying isolates of ST963 sporadically appeared in larger studies related to detection of CMY-2-producers, and *bla*_CMY-2_ was located on plasmids in most instances (5, 7). Only a single study reported evidence for chromosomally encoded *bla*_CMY-2_ in a single human-derived ST963 isolate in Germany (8).

E. coli ST963 has been sporadically reported in wildlife, in companion animals, and very occasionally, in humans (5, 7, 8, 12). It came to our attention as part of an extensive study of E. coli strains with resistance to extended-spectrum beta-lactams and derived from gull chicks nesting on three sites in Australia undertaken in 2012. Whole-genome sequences (WGS) of 425 E. coli isolates (13) show that ST457 (14), ST216 (15), and ST963 were the dominant E. coli sequence types in that study. While our studies suggest that ST457 (14) and ST216 (15) are important emerging E. coli STs, little is known about ST963.

Here, we performed WGS-based comparative analysis of a collection of 111 E. coli ST963 isolates comprising 31 isolates from silver gulls in Australia and 80 GenBank sequences of various source and country origins. The isolates of human and animal origin predominantly originate from Australia and carry *bla*_CMY-2_ on the chromosome. A detailed phylogenetic analysis shed lights on their zoonotic and zooanthropogenic potential.

## RESULTS

### Phylogenetic analyses of E. coli ST963.

**FIG 1:**
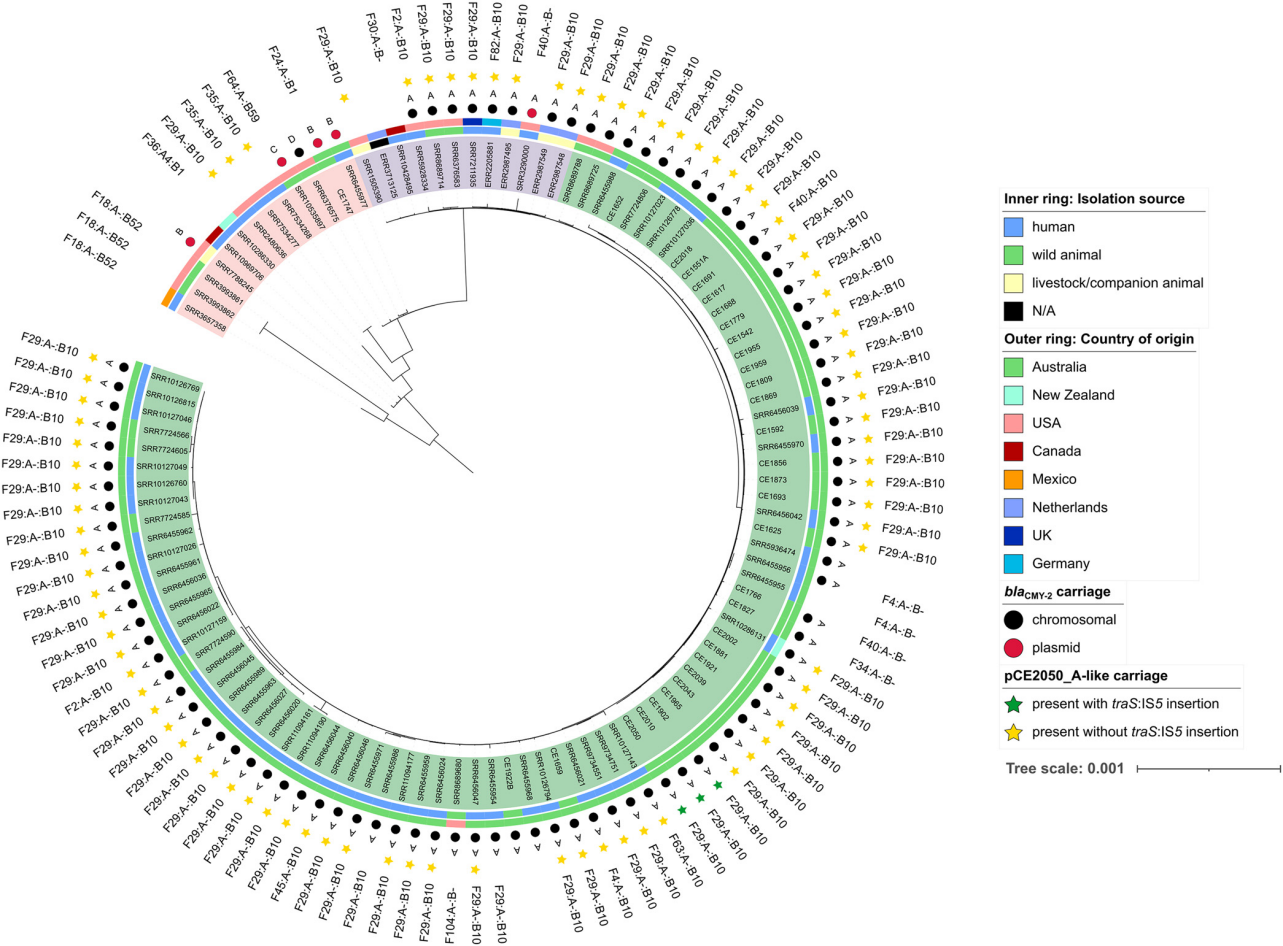
Midpoint rooted phylogenetic tree of the E. coli ST963 collection. The three main clades are highlighted in pink (clade 1), green (“Australian” clade 2), and violet (clade 3). The following information is included: isolation source (inner ring), country of origin (outer ring), chromosomal or plasmid carriage of *bla*_CMY-2_ (black/red circles), variant of genetic surroundings of *bla*_CMY-2_ (characters A to D), carriage of pCE2050_A-like plasmid (green or yellow star, depending on whether the *traS*:IS*5* insertion is present [green] or absent [yellow]), and FAB formulas for F plasmids.

**FIG 2:**
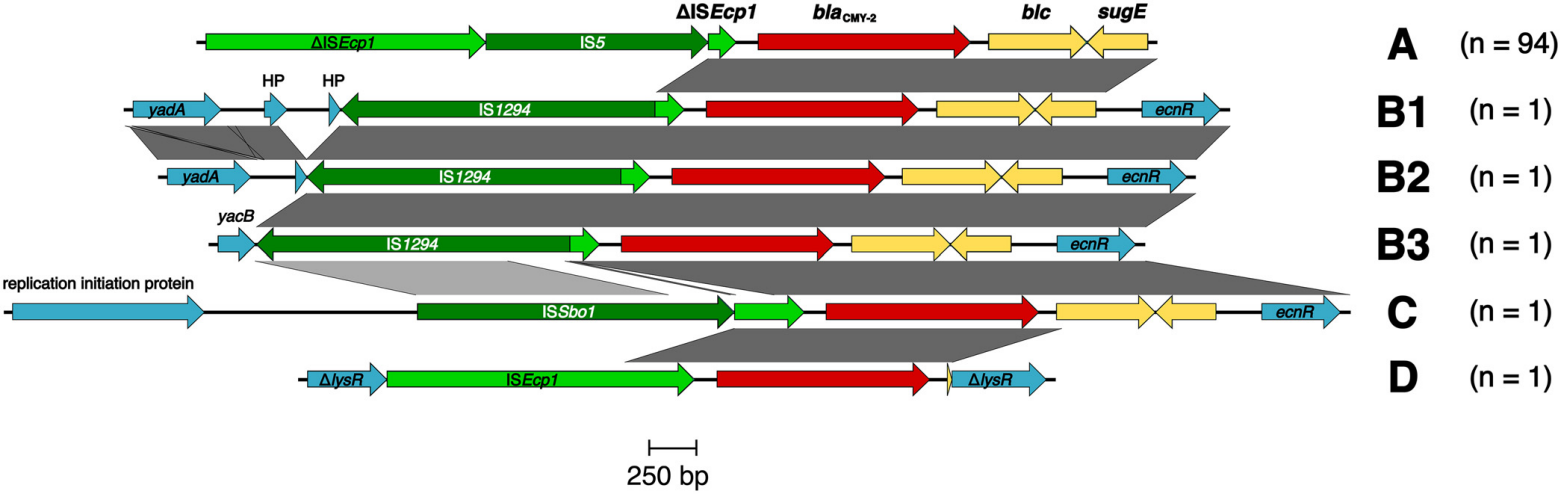
Flanking genomic regions of *bla*_CMY-2_ found within E. coli ST963 strains. Insertion sequences are shown in green, *bla*_CMY-2_ is in red, *blc*, and *sugE* is in yellow; other genes are in cyan. Numbers in parentheses indicate the number of isolates that bear a given genetic surrounding of *bla*_CMY-2_.

A phylogenetic study based on core genome alignments of all 111 ST963 genomes showed a branching pattern consisting of 3 separate clades. A category of evolutionarily distant isolates (*n* = 13) lacking *bla*_CMY-2_ or encoding it on plasmids formed clade 1 (highlighted by pink color in Fig. 1). Only one isolate from clade 1 (GenBank SRA run accession no. SRR6376575) carried a chromosomal copy of *bla*_CMY-2_, but with a genetic context different from remaining isolates that bear chromosomal *bla*_CMY-2_ (variant D, Fig. 2). Isolates from within clade 1 were obtained from all three sources and originated in the United States, Canada, Mexico, New Zealand, and Australia without clear clustering patterns. They all exhibited more than 3,000 single nucleotide polymorphisms (SNPs) against our CE2050 reference sequence. The remaining isolates (*n* = 98) formed a divergent clade which further subdivided into an Australian clade of CMY-2-producing isolates (clade 2, highlighted in green) and another clade of isolates from outside Australia which did not carry *bla*_CMY-2_ as well as isolates carrying *bla*_CMY-2_ on the chromosome or on an I1 plasmid (clade 3), shown in violet in Fig. 1. Clade 2 consisted of 84 isolates, of which 82 were from Australia, 1 was from New Zealand, and the remaining isolate was from the United States (SRR8689680).

To further discriminate between closely related isolates, another tree of 81 isolates with SNP counts below 100 against the CE2050 chromosome was constructed based on SNPs called from mapped sequencing read data (see Fig. S1 in the supplemental material). Of the 81, 43 were obtained from humans, 35 from gulls, and 3 from cows. Most of these closely related isolates originated in Australia (*n* = 76), with the remaining 5 coming from Europe (*n* = 3 from Netherlands, *n* = 1 each from the United Kingdom and Germany). Closely related isolates of animal and human origin, many with less than 20-SNP differences were detected, supporting the hypothesis of their zoonotic and/or zooanthropogenic transmission potential (see “SNP dist” sheet in Table S1). Australian isolates did not appear to cluster according to their territory of origin. Phylogenetic analysis clearly showed that there is a specific CMY-2-producing Australian clone circulating within the country that is spread by humans and animals (humans and gulls were the only available sources of WGS of E. coli ST963 from Australia). The fact that the same transposition unit (variant A) was found on chromosomes of isolates from Germany, Netherlands, United Kingdom, and the United States suggests these isolates shared a recent common ancestor.

10.1128/msphere.00238-22.1 FIG S1 Midpoint rooted phylogenetic tree of 81 closely related E. coli ST963 isolates showing fewer than 100 SNPs against the CE2050 reference chromosome. Branches belonging to clade 2 are highlighted in green, while those belonging to clade 3 are shown in blue. The following information is included: isolation source (inner ring), country of origin (outer ring), chromosomal or plasmid carriage of *bla*_CMY-2_ (black/red circles), variant of genetic surroundings of *bla*_CMY-2_ (characters A to D), and carriage of pCE2050_A-like plasmid (green or yellow star, depending on whether *traS*:IS*5* insertion is present [green] or absent [yellow]). Download FIG S1, TIF file, 0.9 MB. Copyright © 2022 Medvecky et al. 2022 Medvecky et al. https://creativecommons.org/licenses/by/4.0/ This content is distributed under the terms of the Creative Commons Attribution 4.0 International license.

10.1128/msphere.00238-22.3 TABLE S1 The overview of E. coli ST963 genomes, including the metadata used for the comparative analysis. (A) The following information is included: raw SNP counts (*), refined SNP counts (**), and presence/absence of the pCE2050_A-like plasmid (≥90% coverage of pCE2050_A) (***). (B) Results of antibiotic susceptibility profiles are interpreted as resistant in red, intermediate in blue, and susceptible in green. Antibiotic abbreviations include AMP, ampicillin; S, streptomycin; S3, sulphonamides; TE, tetracycline; SXT, sulphamethoxazole-trimethoprim; C, chloramphenicol; KZ, cefazolin; NA, nalidixic acid; CAZ, ceftazidime; CN, gentamicin; AMC, amoxicllin-clavullanic acid; CIP, ciprofloxacin; FOS, fosfomycin; ETP, ertapenem; IPM, imipenem; MEM, meropenem; ATM, aztreonam; F, nitrofurantoin; AZM, azithromycin; and TGC, tigecyclin. (C) *: FQ, fluoroquinolones. (C) FQ stands for fluoroquinolones (*) and ARG for antibiotic resistance gene (**). The presence of the genes is shown in green (≥60% coverage and ≥90% sequence identity). (D) The presence of the gene of interest is shown by colored boxes as follows: present (≥80% coverage and/or multiple copies), partially present (≤40 to  <80% of coverage), partially present (≤10 <40% of coverage). IS, insertion sequences; VAG, virulence-associated genes. (E) Thresholds of ≥95% sequence identity and ≥60% of coverage were used. (F) pMLST, plasmid multilocus sequence typing; ST, sequence type; CC, clonal complex. Download Table S1, XLSX file, 0.1 MB. Copyright © 2022 Medvecky et al. 2022 Medvecky et al. https://creativecommons.org/licenses/by/4.0/ This content is distributed under the terms of the Creative Commons Attribution 4.0 International license.

### Genomic features of a reference genome of E. coli ST963.

Our closed reference genome CE2050 comprised a chromosome of 4,979,906 bp and two plasmids, pCE2050_A, a 115,443-bp F29:A-:B10 plasmid, and pCE2050_B, a 222,008-bp IncHI2 plasmid typed as ST3. While pCE2050_A was carried by most E. coli ST963 isolates described in this study (see below, Fig. 1), pCE2050_B was only present in isolate CE2050. One intact prophage region was identified on the chromosome (nucleotides [nt] 2050804 to 2082987 in GenBank accession no. CP073621), showing a high level of similarity to prophage PHAGE_Entero_WPhi_NC_005056. There was also cephalosporinase gene *bla*_CMY-2_ found on the chromosome along with 10 different virulence factors (see Table S1). Plasmid pCE2050_A encodes Col156, IncFIB, and IncFII plasmid replicons, and its replicon sequence type (RST) is F29:A-:B10. The same plasmid ST is a reliable predictor of plasmids that bear resemblance to virulence plasmid pUTI89 (16). These two plasmids are nearly identical in length (115,443 bp versus 114,230 bp, respectively) and gene content and exhibit few SNPs. Similar to other pUTI89-like plasmids, pCE2050_A did not carry any antibiotic resistance gene (ARGs); however, it carried an IS*26* and a virulence factor, *traT.* Notably, pCE2050_A carried a *traS*:IS*5* sequence (nt 6770 to 7964 in pCE2050_A) absent from pUTI89, flanked by a CTAA 4-bp direct repeat (DR) on either end of the IS*5* element. pCE2050_B is closely related to other IncHI2 plasmids, including pCE1681-A (94% coverage and 100% identity) typed as ST3, which was carried by E. coli ST216 recovered from a silver gull in Australia (GenBank accession no. MT180430) (15) in 2012. pCE2050_B harbored a 1,131-bp fragment that shows similarities to sequences previously described in IncN plasmids (17). This fragment included the *repA* gene and a part of the iteron region. pCE2050_B carries an MDR region with a Tn*1721*-specific tetracycline module with the *tet*(A) gene inserted in the same site as in other IncHI2 plasmids (18) and an In369 class 1 integron with *dfrA1b* and *aadA1b* gene cassettes.

### Antibiotic resistance, virulence genes, and plasmids of the ST963 collection.

All ST963 isolates from our Australian gull collection (*n* = 31) were resistant to ampicillin, cefazolin, ceftazidime, and amoxicillin, which is in accordance with their AmpC phenotype. They all also exhibited resistance or intermediate susceptibility to aztreonam and streptomycin. Analysis of this gull collection, and an additional 80 ST963 WGS data sets from EnteroBase, showed that *bla*_CMY-2_ was present in all isolates from our Australian silver gull collection (31/31) and in 85% of EnteroBase sequences (68/80). *bla*_CMY-2_ was identified in 52 out of 60 isolates from clinical samples of humans, 43/45 isolates obtained from wild animals, and 4 of 5 isolates from livestock/companion animals. Other resistance genes were present in much lower frequencies. The second most prevalent antibiotic resistance gene (ARG) in the collection was *bla*_TEM-1B_ (encoding narrow-spectrum β-lactamase) found in 10 of 111 isolates, followed by genes for resistance to sulfonamides, *sul2* (9/111) and *sul1* (8/111), and aminoglycosides, *aph(3′')-Ib* (7/111) and *aph(6)-Id* (7/111). A complete list of predicted ARGs can be found in Table S1. On average, isolates carried 1.7 ARGs, indicating that while E. coli ST963 frequently harbors *bla*_CMY-2_, genotypic MDR is unusual. IS*26* (99/111; 89%) was frequently identified in the collection. A total of 14 (13%) isolates carried the *intI1* gene for class 1 integrase.

Virulence gene analysis showed that all isolates harbored *fimH*, *fyuA*, *irp2*, and *silA* on the chromosome and several copies of *ipaH and yeeT* located on the chromosome as well. There was also high prevalence of chromosomally encoded *sitA* (*n* = 107) and plasmid-carried *traT* (*n* = 101) among the isolates under investigation, while other putative virulence factors were present in lower frequencies. A complete list of virulence-associated genes (VAGs) is given in Table S1.

Most (88/111; 79%) ST963 isolates, which included representation from humans (48/60; 80%) and wildlife (34/45; 76%) as well as livestock/companion animals (4/5), carried either the 115,443- bp F plasmid pCE2050_A or closely related variants with more than 90% of the pCE2050_A sequence. Analysis of all 88 pCE2050-like bearers showed that the Δ*traS*:IS*5* insertion was only present in three closely related isolates (CE2050, CE2010, and CE1902, all showing ≤3 SNP differences when compared to each other). pCE2050_A showed 99% coverage and 99.95% identity with a pUTI89-like plasmid named XXX from the GenBank database (accession no. LR730401) that was carried by E. coli ST73 from vasculated human blood in Germany. Apart from pCE2050_A replicons, PlasmidFinder analysis showed another 29 FII replicons, followed by 17 I1 and 15 FIB replicons that were present within the studied sample set. The FAB formulas for F plasmids along with a complete list of plasmid replicons that were identified in the study can be found in Table S1.

### Genetic context of *bla*_CMY-2_.

Of the 89% (99/111) of ST963 which carry *bla*_CMY-2_, the vast majority (94/99) had the gene located chromosomally (Fig. 1). In the remaining five isolates, it was carried by various IncI1-I(α) plasmids, which belonged to clonal complex 2 (CC2; two had ST2, two had ST23) or ST19. Two I1/ST23 plasmids, including the 92,387-bp pCE1747_I1 from our silver gull collection and the 89,653-bp pCH12 from a human (GenBank SRA run accession no. SRR6455977), originated in Australia and were closely related to the 94,698-bp plasmid pCE1628_I1 (14) from E. coli ST457 (GenBank accession no. MT468651), isolated from a silver gull in Australia, suggesting that plasmid sharing among E. coli STs occurs in gull colonies. pCH12 shows 100% sequence coverage and 99.98% sequence identity with pCE1628_I1, while pCE1747_I1 exhibited 97% coverage and 99.97% identity with pCE1628_I1 using BLASTn. I1/ST2 plasmid pECOL-18-VL-SD-IA-0003 (88,569 bp) from a canine source in the United States (GenBank SRA run accession no. SRR7788245) shows 99% coverage and 99.97% sequence identity with an 88,229-bp pAR-0430-1 (GenBank accession no. CP044137) from an E. coli O157 isolate with ST11. Metadata regarding the source of isolation or country of origin is unavailable. An I1/ST19 plasmid with an approximate size of 94 kbp (GenBank SRA run accession no. SRR3290000) obtained from a human in the United States shows high similarity to the 94,170-bp plasmid p4540-1 (GenBank accession no. CP041533) recovered from E. coli ST963 from an unrelated human in the United States. Using BLASTn, the I1/ST19 plasmid exhibited 97% sequence coverage and 99.68% nucleotide identity of with p4540-1. The remaining I1/ST2 plasmid pECOL-19-VL-WA-KY-0014, recovered from a wolf in the United States (GenBank SRA run accession no. SRR10535897), showed 100% sequence coverage and 99.99% sequence identity with the 94,881-bp plasmid p95 (GenBank accession no. CP023356) carried by an E. coli typed as ST963 that was obtained from a canine source in the United Kingdom in 2002. Metadata relating to isolates carrying *bla*_CMY-2_-positive plasmids can be found in Table S1.

### Analysis of regions flanking *bla*_CMY-2_.

*bla*_CMY-2_ was identified in four different genetic environments in the chromosome of ST963, here referred to as variants A to D (Fig. 2). Most (94/111; 85%) isolates, including those from humans, wildlife and livestock, and companion animals (51/60, 40/45, and 3/5, respectively), carried a similar genetic arrangement found on the chromosome of C. freundii (*bla*_CMY-2_-*blc*-*sugE*) (19). The transposition unit was likely mobilized by IS*Ecp1* (IS*1380* family) truncated by insertion of an IS*5* element (Fig. 2A). The vast majority of isolates carried *bla*_CMY-2_ as part of a transposition unit defined here as variant A on the chromosome (*n* = 93). Variant A was mostly located within the *yehB* gene, which is part of the *yehABCD* fimbrial operon, and was flanked by a TATAA DR. An identical transposition unit was also present on an I1/ST19 plasmid from a human isolate from our EnteroBase E. coli ST963 collection originating in the United States (GenBank SRA run accession no. SRR3290000). BLASTn analysis of the 3,931-bp-long region comprising ΔIS*Ecp1*-IS*5*-ΔIS*Ecp1*-*bla*_CMY-2_ (nt 1417762 to 1421692 in GenBank accession no. CP073621) showed only four high-scoring hits, all of them having a *bla*_CMY-2_-*blc*-*sugE* module. Two hits belonged to E. coli ST963 chromosomal sequences (GenBank accession no. CP051733 and LR130562), one was to I1/ST19 plasmid p4540-1 (GenBank accession no. CP041533) from E. coli ST963 obtained in 2012 from infected human blood in the United States, and the remaining match belonged to IncC-ST3 plasmid p25358-2 (GenBank accession no. CP051443) from Salmonella enterica (20) isolated in 2002 from turkey in the United States.

B variants were identified in three isolates from a human, a silver gull, and a canine, all three of which carried *bla*_CMY-2_ on different IncI1-I(α) plasmids. In each of these isolates a copy of IS*1294* followed by a small, truncated portion of IS*Ecp1* was identified downstream of *bla*_CMY-2_. Variant B showed similarity to a region found in plasmid pS10584 from Salmonella enterica from China (GenBank accession no. KX058576). Various B variants differed from each other in the flanking region upstream of the IS*1294*-ΔIS*Ecp1*-*bla*_CMY-2_-*blc*-*sugE* module (Fig. 2). Variant C was identified in a single ST963 isolate from a wolf in the United States (GenBank SRA run accession no. SRR10535897). Here, *bla*_CMY-2_ was detected on another IncI1-I(α) plasmid. In variant C, IS*Sbo1* (an IS*91* family element) followed by a truncated portion of IS*Ecp1* was located upstream of *bla*_CMY-2_. Direct repeats indicating transposition of the *bla*_CMY-2_-carrying unit into the specific genetic element were not detected. A single isolate obtained from a wild bird (GenBank SRA run accession no. SRR6376575) carried a chromosomally localized *bla*_CMY-2_ gene also associated with IS*Ecp1* (variant D). *bla*_CMY-2_ along with small portion of *blc* was inserted into *lysR* and was flanked by TATTA DRs. Metadata associated with these genomic assemblies and their *bla*_CMY-2_-carrying transposition units is available in Table S1.

### Closely related ST963 strains are shared by humans and gulls.

ST963 isolates are phylogenetically diverse; nearly three quarters (81/111; 73%) of all ST963 genomes displayed fewer than 100 SNPs compared to the CE2050 chromosome, while 13% (14/111) showed more than 1,000 SNPs against our reference chromosome. Isolates with more than 3,000 SNPs compared to the CE2050 chromosome reside in clade 1 (shown in pink color in Fig. 1). The remaining ST963 isolates represented a dominant clade that was further divided into two subclades (clade 2, shown in green and clade 3, shown in violet). In clade 2 the E. coli ST963 genomes, including those from gulls from three sites and from humans, contained most (at least 98.5%) of the CE2050 chromosome. Interestingly, most ST963 isolates with >1,000 SNP differences from the reference chromosome either did not carry *bla*_CMY-2_ (8/14; 57%) or *bla*_CMY-2_ was located on a plasmid (4/14; 29%). Plasmid-mediated *bla*_CMY-2_ was detected in samples originating in all three source categories (human, wildlife, and livestock/companion animals), namely, human, gull, wolf, and dog. There were only two isolates with >1,000 SNPs that encoded *bla*_CMY-2_ in the chromosome. An isolate with 1,154 SNPs, obtained from a human in Australia (SRR11094161), carried a variant A *bla*_CMY-2_ genetic arrangement (Fig. 1, clade 2). Another isolate, with 3,190 SNPs, isolated from a bald eagle in the United States (SRR6376575), carried a variant D *bla*_CMY-2_ genetic context (Fig. 1, clade 1).

The cohort was separated into two groups, one of close relatives (defined as ≤100 SNPs against the CE2050 chromosome and referred to as “group1”) and one of more distant relatives (defined as >100 SNPs, referred to as “group2”). Site-wise aggregation of SNP counts was undertaken and visualized (see Fig. S2). The distribution of SNPs within group1 appeared uniform except for a highly variable region which contained over 400 SNPs (Fig. S2A). Most of those SNPs were located in a sequence encoding GTPase Era, which was found as a family comprising four paralogs in the CE2050 chromosome. Due to possible mis-alignment of reads originally belonging to other copies of Era genes, SNPs found in this region were discarded from the alignment file used for the construction of a detailed phylogeny inference (Fig. S1). When scrutinizing group2 genomes, four regions each with an elevated number of SNPs were observed. Detailed genomic information about these regions can be found in the Table S3.

10.1128/msphere.00238-22.2 FIG S2 Histograms showing site-wise aggregation of SNP counts. (A) A group of isolates with ≤100 SNPs against the CE2050 chromosome. (B) A group of isolates (B) with >100 SNPs against the CE2050 chromosome. Download FIG S2, TIF file, 0.2 MB. Copyright © 2022 Medvecky et al. 2022 Medvecky et al. https://creativecommons.org/licenses/by/4.0/ This content is distributed under the terms of the Creative Commons Attribution 4.0 International license.

10.1128/msphere.00238-22.4 TABLE S2 Custom database of ExPEC virulence gene sequences. Download Table S2, XLSX file, 0.1 MB. Copyright © 2022 Medvecky et al. 2022 Medvecky et al. https://creativecommons.org/licenses/by/4.0/ This content is distributed under the terms of the Creative Commons Attribution 4.0 International license.

All ST963 isolates (*n* = 81) exhibiting fewer than 100 SNPs to the reference CE2050 chromosome, as well as the reference strain itself, carried *bla*_CMY-2_ on the chromosome, except for one isolate where we identified a single SNP that led to a change in the *bla*_CMY_ allele to *bla*_CMY-143_. Chromosomally encoded *bla*_CMY-2_ was present in most isolates (11/15; 73%) with SNP counts in the range of 100 to 1,000 (isolates mostly from clade 3, shown in violet color in Fig. 1). All isolates with SNP counts below 1,000 that carried *bla*_CMY-2_ on the chromosome carried variant A (see Fig. 1). We also noticed a correlation between chromosomal SNP counts and carriage of F plasmid pCE2050_A (or its close variants) within ST963 genomes. While they were identified in 79% (88/111) of ST963 isolates in total, these closely related plasmids were carried by 88% (72/82) of isolates with fewer than 100 SNPs to the reference, by 80% (12/15) isolates exhibiting SNP counts in the range of 100 to 1,000, and only by 29% (4/14) of isolates showing more than 1,000 SNPs.

## DISCUSSION

ST963 is a phylogroup D E. coli strain closely related to ST38 in CC 38. At the time of writing (May 2022), a search of the PubMed database matches did not return a hit to the term “E. coli ST963,” highlighting the lack of information on this sequence type. Nonetheless, ST963 has been reported in studies of E. coli recovered from wild and urban-adapted birds (12, 21). Here, we characterized 111 ST963 isolates originating in humans (*n* = 60) and wildlife (*n* = 45 [31 of which are from Australian silver gulls]), as well as livestock/companion animals (*n* = 5). The isolates were recovered between 1984 and 2019 and predominantly sourced from Australia (*n* = 89). While we contributed 31 E. coli ST963 strains from Australian silver gulls, the remaining 80 E. coli ST963 genomes were from geographically dispersed regions that were deposited in EnteroBase (http://enterobase.warwick.ac.uk/).

All E. coli ST963 genomes harbored multiple VAGs, including *fimH*, *fyuA*, *irp2*, *silA*, *ipaH*, and *yeeT*, and many also carried *sitA*, *traT*, and the insertion element IS*26.* Cocarriage within each isolate of iron-acquisition genes *fyuA* and *irp2* suggests the presence of the *Yersinia* high-pathogenicity island, a chromosomal genomic island associated with increased capacity for avian infection and uropathogenesis (22). *senB*, a virulence gene used as a marker gene for the presence of virulence plasmid pUTI89, was indeed present in all isolates (*n* = 88) that carried pUTI89-like plasmid pCE2050_A or its close variants covering more than 90% of its sequence. Carriage of the *bla*_CMY-2_-carrying AmpC β-lactamase CMY-2 was a feature of ST963. It was identified in all isolates from our Australian silver gull collection as well as in 85% (*n* = 68/80) of ST963 sequences from EnteroBase, irrespective of source, suggesting that *bla*_CMY-2_ is strongly associated with globally dispersed E. coli ST963.

Overall, E. coli ST963 exhibited low carriage of ARGs (1.7 on average), in contrast with closely related E. coli ST38 (from the same clonal complex). E. coli ST38 strains obtained from infected humans frequently carry multiple ARGs conferring resistance to first-line antibiotics and, in some cases, genes encoding ESBLs (23, 24). Notably, *bla*_CMY-2_ was predominantly encoded on the chromosome, with 93 isolates showing the genetic context surrounding *bla*_CMY-2_ similar to that reported in the chromosome of C. freundii (*bla*_CMY-2_-*blc*-*sugE*). The transposition unit carrying *bla*_CMY-2_-*blc*-*sugE* appears to have been initially mobilized by IS*Ecp1* but has subsequently been infiltrated by the insertion of IS*5*, creating a unique trackable signature. Insertion of IS*5* into the coding sequence of IS*Ecp1* could explain the inactivation of the insertion element IS*Ecp1*, which has been originally associated with the mobilization of the *bla*_CMY-2_ resistance gene. The fact that the insertion site of the *bla*_CMY-2_-carrying genetic determinant was identical in the majority of the studied isolates further supports the above-described hypothesis. Insertion elements are known to alter the expression of clinically important ARGs. In this regard, IS*Ecp1* is not only involved in gene mobilization, but it also enhances expression of *bla*_CTX-M_ β-lactamases (25). IS*5* was previously associated with increased *bla*_CMY-2_ expression through a tandem gene amplification mechanism described in clinical E. coli strains (26). Overall low carriage of ARGs along with frequent presence of *bla*_CMY-2_ within E. coli ST963 genomes suggests that this E. coli lineage is possibly early in its evolutionary trajectory as a potential human antimicrobial resistance threat.

All ST963 genomes that were closely related (<100 SNPs) to our reference genome CE2050 harbored a chromosomal copy of *bla*_CMY-2_. *bla*_CMY-2_ was also present on the chromosome of most isolates (11/15; 73%) with SNP counts against CE2050 chromosome between 100 and 1,000, suggesting that its location on the chromosome is ancestral. In contrast, isolates divergent from CE2050 (>1,000 SNPs) either lack *bla*_CMY-2_ or it was located on various I1 plasmids. Only two such divergent isolates encoded *bla*_CMY-2_ chromosomally. One carried our predominant transposition unit (variant A), while another isolate carried variant D, and as such, it contained an intact copy of IS*Ecp1*and lacked IS*5* as well as large portion of *blc* and all of *sugE*, probably due to further mobilization events that occurred after the initial transposition unit was incorporated into the chromosome. These data highlight some of the ongoing evolutionary events that shape carriage of *bla*_CMY-2_ in ST963.

We also noted a correlation between chromosomal SNP counts and carriage of the 115,443-bp F plasmid pCE2050_A from our closed reference genome. pCE2050_A and variants of it were borne by 87% of isolates with SNP counts below 1,000 compared to the CE2050 chromosome, while it was present only in 29% of isolates that showed more than 1,000 SNPs against our reference. pCE2050_A is an F29:A-:B10 with high nucleotide identity with pUTI89. However, it is notable that pCE2050_A carries a *traS*:IS*5* insertion absent from pUTI89. The truncation of the Tra region by IS*5* could result in the loss of the conjugation ability of pCE2050_A. This insertion event was not detected within the NCBI nucleotide collection; however screening our collection identified it in three pCE2050_A-like bearers that were closely related to each other. This suggests a recent incorporation of the IS*5* element into pUTI89, potentially enabling further mobilization of *bla*_CMY-2_ from the chromosome/transposition unit to pUTI89 via homologous recombination. This insertion sequence and its junction with *traS* therefore constitute a distinct, trackable genetic motif for pCE2050_A and related plasmids.

pUTI89 is a globally dispersed F virulence plasmid with an F29:A-:B10 replicon type found in several top 20 E. coli STs causing clinical disease (27). pUTI89 was first reported in strain UTI89, an ST95 E. coli from a patient with an acute bladder infection (28), and has been assessed for its ability to cause disease in a mouse urinary tract infection (UTI) infection model (29, 30). Specifically, pUTI89 and close variants of it have been found in some but not all sublineages of ST95 (27, 31), ST127 (32), and ST131 (33), E. coli STs that are dominant clinical uropathogens (11, 34). Despite a copy of IS*26*, a key element involved in the rearrangement and spread of multiple ARGs, being present in pUTI89, these plasmids rarely carry ARGs; however, they harbor important virulence factors, including the *cjrABC*-*senB* operon and *traT* gene.

We did not observe any correlation between carriage of pCE2050_A-like plasmids and source of isolation. Our closed reference genome CE2050 also contained a 222,008-bp IncHI2-N plasmid, pCE2050_B, that consisted of an IncHI2 plasmid backbone and a 1,131-bp fragment showing similarities to sequences previously described in IncN plasmids. pCE2050_B is similar to HI2 plasmid pCE1681-A (15) obtained from E. coli ST216 from our Australian gull chick nesting collection. Strain CE2050 was recovered from Montague Island (also known as Barunguba Island), while ST216 strain CE1681, which bears a very similar HI2 plasmid, was sourced from Five Islands Nature Reserve. These two islands are separated by approximately 200 km along the eastern coastline of New South Wales, Australia.

Our analyses revealed significant variability in SNP counts (3 to 11,273 SNPs) when sequencing reads were mapped against the CE2050 chromosome. Isolates with <100 SNPs showed a generally uniform distribution of SNPs relative to the reference CE2050 genome, while SNP frequency distribution in isolates exhibiting more than 100 SNPs revealed four SNP hot spot regions within approximately 300 kbp (Fig. S2A and B). Those were found mainly in divergent isolates carrying more than 1,000 SNPs against our reference genome. This extensive SNP heterogeneity of E. coli ST963 strains suggests the need for the employment of typing methods with higher discriminatory power than standard multilocus sequence typing (MLST) schemes have, such as core genome MLST (cgMLST) or even the reference-independent phylogenetic approach to avoid reference bias.

In summary, phylogenetic analyses identified a unique specific clade of Australian ST963 isolates harboring a chromosomal copy of *bla*_CMY-2_. This Australian clade comprised phylogenetically close clusters (<20 SNP differences from the reference genome) of isolates of both animal and human origin, providing evidence of interspecies transmission. The fact that isolates originating outside Australia segregate to different clades, separated from the major Australian clade, supports the hypothesis of the emergence of a specific *bla*_CMY-2_-carrying E. coli ST963 clone in Australia that is widely spread across the continent by humans and birds. The presence of transposition unit ΔIS*Ecp1*-IS*5*-ΔIS*Ecp1*-*bla*_CMY-2_-*blc*-*sugE*, inserted into the same location within the chromosome of isolates from Australia, Germany, Netherlands, United Kingdom, and the United States point to a shared recent common ancestor.

## MATERIALS AND METHODS

### Strain collection.

The collection examined here comprised 35 E. coli isolates cultivated on MacConkey agar with cefotaxime (2 mg/L) from cloacal swabs from silver gulls (Chroicocephalus novaehollandiae) that were collected in three nesting colonies in New South Wales (NSW), Australia, in 2012 (35) and subjected to Illumina sequencing. Illumina WGS data of a further 81 E. coli ST963 strains were gathered from the GenBank SRA database based on information found in EnteroBase (http://enterobase.warwick.ac.uk; the search was conducted in February 2020). In total, there were 60 sequences originating from human clinical isolates, 50 from wild animals, 5 from livestock/companion animals, and a single isolate with missing information regarding the source. Isolates were collected between 1984 and 2019; however, most strains were collected in 2012 or later (*n* = 102); three isolates lacked the year of isolation. Countries of origin included Australia (*n* = 89), the United States (*n* = 16), Netherlands (*n* = 4), Canada (*n* = 2), New Zealand (*n* = 2), the United Kingdom (*n* = 1), Germany (*n* = 1), and Mexico (*n* = 1). After an initial quality check, duplicate genomes (*n* = 5; 4 isolates from our gull collection and a single gull isolate from EnteroBase) were discarded from downstream analyses, leaving 111 samples in the final data set. Detailed metadata are available in Table S1.

### Antibiotic susceptibility testing, DNA extraction, and sequencing.

The disk diffusion methodology with a set of 21 antibiotics (Oxoid, Hants, UK) was performed according to European Committee on Antimicrobial Susceptibility Testing (EUCAST) recommendations (36). Inhibition zone diameters of the tested isolates were measured and interpreted according to EUCAST breakpoints (36) or using breakpoints defined by CLSI in 2017 for antibiotics (azirthromycin, cefazolin, tetracycline, nalidixic acid, sulfonamide compounds, and streptomycin) with no defined breakpoints in EUCAST (36, 37). Susceptibility to colistin was accessed using the Colispot test (38).

The production of ESBL/AmpC enzymes was assessed using a D68C1 AmpC and ESBL detection set (Mast Diagnostics, UK). Results of susceptibility testing for all tested antibiotics and their interpretation can be found in Table S1. Genomic DNA was extracted and purified using NucleoSpin columns (NucleoSpin tissue; Macherey-Nagel, Germany). Fragment libraries were constructed using Nextera XT kits followed by paired-end sequencing (NovaSeq; Illumina) according to the manufacturer’s instructions. For the E. coli CE2050 strain, long-read sequencing was performed on the Sequel I platform (Pacific Biosciences, Menlo Park, CA, USA). A microbial multiplexing protocol was used for the library preparation according to the manufacturer’s instructions for sheared DNA. DNA shearing was performed using Covaris g-TUBES (Covaris, USA). No size selection was performed during the library preparation.

### Assembly of sequencing reads.

Raw Illumina sequencing reads were trimmed using Trimmomatic v0.36 (39), assembled with SPAdes v3.12.0 (40), and polished with Pilon v1.23 (41).

Plasmids carrying *bla*_CMY-2_ were scaffolded by mapping contigs to the respective reference sequences from GenBank, which were chosen based on BLASTn (42) alignments of *bla*_CMY-2_-positive contigs against standard nonredundant nucleotide collection. QUAST v5.0.2 (43) was then used for the analysis of whole-genome data aligned to selected candidate reference sequences in order to ensure that they were fully covered. Contig overlaps were manually inspected via Tablet v1.20.12 (44) by visualization of Illumina reads mapped against overlapping regions. Locations with uncertain connections were indicated by 100 N characters.

For isolate CE2050, for which long-read sequencing data were generated, the assembly and circularization of PacBio reads was performed using the Microbial Assembly pipeline offered by the SMRT Link v8.0 software using minimum seed coverage of 30×. The closed genome was polished with Pilon v1.23 (41).

### Comparative analysis, SNP calling, and phylogenetics.

The assembled data were annotated using Prokka v1.14.1 (45) and analyzed for the presence of antimicrobial resistance genes and plasmid replicons using ResFinder (46) and PlasmidFinder (47), respectively. *bla*_CMY-2_-carrying I1 plasmid sequences as well as F plasmid replicons were subjected to plasmid MLST (pMLST) and replicon sequence typing (RST) analysis, respectively (47). Genomes were also checked for the presence of virulence-associated genes using our custom database of ExPEC virulence gene sequences (see Table S2). Genomes were interrogated for the presence of prophage regions via PHASTER (48), and insertion sequences, via ISfinder (49). Initial comparative analysis was conducted using QUAST v5.0.2 (43) with manual inspection of genomic discrepancies via Icarus (50). SNP hot spot regions were annotated using the RAST annotation pipeline, and predicted genes were functionally categorized with SEED (51).

The assembled genomes were screened for the presence of pUTI89-like plasmids by mapping contigs against the closed reference plasmid pCE2050_A from strain CE2050. The presence of the *traS*:IS*5* sequence in pUTI89-like plasmids within our collection was then investigated manually by visual checks of mapped sequencing reads against the pCE2050_A reference via Tablet v1.20.12 (44). The Burrows-Wheeler Aligner (BWA) MEM algorithm (52) was used for contig as well as read mapping.

Quality-trimmed Illumina reads were mapped to the closed chromosome of CE2050 using Bowtie 2 v2.3.4.2 (53). SNPs were then detected with VarScan v2.4.3 (54) using the following parameters: minimum read depth of 8, minimum base quality of 20, variant allele frequency of ≥0.8. Variant sites occurring in phage and repetitive/homologous regions as well as sites in which at least one sample had a read depth below 8 were discarded from subsequent phylogenetic analysis. SNP distances among samples were calculated with the snp-dists tool v0.7.0 (https://github.com/tseemann/snp-dists). Based on called SNP sites, QUAST metrics and PlasmidFinder results, several samples (three pairs and a single trio) were considered identical. Duplicate genomes (*n* = 5; 4 isolates from our gull collection and a single gull isolate from EnteroBase) were discarded.

Filtered SNP sites from isolates with fewer than 100 SNP (*n* = 81) differences from the CE2050 chromosome were concatenated and analyzed using jModelTest v2.1.10 (55) to estimate the best-fitting model of nucleotide substitution. Using Akaike as well as Bayesian criteria, the general time-reversible (GTR) substitution model was determined as the best fit and therefore used for the maximum-likelihood (ML) analysis. ML tree topology was then inferred using RAxML v8.2.10 (56) with 500 rapid bootstrap replicates. A phylogenetic tree of all E. coli ST963 isolates was constructed based on a core genome determined using the Roary pipeline v3.12.0 (57) and aligned with MAFFT v7.313 (58). Tree topology was inferred via FastTree v2.1.11 (59), which was compiled with double-precision arithmetic. Both trees were visualized using iTOL v6.1.1 (60) and edited in Inkscape v0.92 (www.inkscape.org).

### Data availability.

A total of 31 SRA archives of our isolate collection are deposited in GenBank under BioProject PRJNA630096. Our closed reference genome CE2050 along with draft assemblies of other isolates from our collection are deposited under BioProject PRJNA723472.

10.1128/msphere.00238-22.5 TABLE S3 Genomic information of E. coli ST963 regions with elevated numbers of SNPs. Download Table S3, XLSX file, 0.01 MB. Copyright © 2022 Medvecky et al. 2022 Medvecky et al. https://creativecommons.org/licenses/by/4.0/ This content is distributed under the terms of the Creative Commons Attribution 4.0 International license.

## References

[1] Pfeifer Y, Cullik A, Witte W. 2010. Resistance to cephalosporins and carbapenems in gram-negative bacterial pathogens. Int J Med Microbiol 300:371–379. doi:10.1016/j.ijmm.2010.04.005. 20537585

[2] Denisuik AJ, Lagace-Wiens PRS, Pitout JD, Mulvey MR, Simner PJ, Tailor F, Karlowsky JA, Hoban DJ, Adam HJ, Zhanel GG, Zhanel GG, Hoban DJ, Adam HJ, Karlowsky JA, Baxter MR, Nichol KA, Lagace-Wiens PRS, Walkty A, on behalf of the Canadian Antimicrobial Resistance Alliance (CARA). 2013. Molecular epidemiology of extended-spectrum β-lactamase-, AmpC β-lactamase- and carbapenemase-producing Escherichia coli and Klebsiella pneumoniae isolated from Canadian hospitals over a 5 year period: CANWARD 2007–11. J Antimicrob Chemother 68:i57–i65. doi:10.1093/jac/dkt027. 23587779

[3] Bauernfeind A, Stemplinger I, Jungwirth R, Giamarellou H. 1996. Characterization of the plasmidic beta-lactamase CMY-2, which is responsible for cephamycin resistance. Antimicrob Agents Chemother 40:221–224. doi:10.1128/AAC.40.1.221. 8787910 PMC163087

[4] Kang MS, Besser TE, Call DR. 2006. Variability in the region downstream of the blaCMY-2 beta-lactamase gene in Escherichia coli and Salmonella enterica plasmids. Antimicrob Agents Chemother 50:1590–1593. doi:10.1128/AAC.50.4.1590-1593.2006. 16569893 PMC1426964

[5] Naseer U, Haldorsen B, Simonsen GS, Sundsfjord A. 2010. Sporadic occurrence of CMY-2-producing multidrug-resistant Escherichia coli of ST-complexes 38 and 448, and ST131 in Norway. Clin Microbiol Infect 16:171–178. doi:10.1111/j.1469-0691.2009.02861.x. 19548922

[6] Baudry PJ, Mataseje L, Zhanel GG, Hoban DJ, Mulvey MR. 2009. Characterization of plasmids encoding CMY-2 AmpC beta-lactamases from Escherichia coli in Canadian intensive care units. Diagn Microbiol Infect Dis 65:379–383. doi:10.1016/j.diagmicrobio.2009.08.011. 19775846

[7] Hansen KH, Bortolaia V, Nielsen CA, Nielsen JB, Schønning K, Agersø Y, Guardabassi L. 2016. Host-specific patterns of genetic diversity among IncI1-Igamma and IncK plasmids encoding CMY-2 beta-lactamase in Escherichia coli isolates from humans, poultry meat, poultry and dogs in Denmark. Appl Environ Microbiol 82:4705–4714. doi:10.1128/AEM.00495-16. 27235431 PMC4984282

[8] Pietsch M, Irrgang A, Roschanski N, Brenner Michael G, Hamprecht A, Rieber H, Käsbohrer A, Schwarz S, Rösler U, Kreienbrock L, Pfeifer Y, Fuchs S, Werner G, RESET Study Group. 2018. Whole genome analyses of CMY-2-producing Escherichia coli isolates from humans, animals and food in Germany. BMC Genomics 19:601. doi:10.1186/s12864-018-4976-3. 30092762 PMC6085623

[9] Ambrose SJ, Harmer CJ, Hall RM. 2018. Compatibility and entry exclusion of IncA and IncC plasmids revisited: IncA and IncC plasmids are compatible. Plasmid 96–97:7–12. doi:10.1016/j.plasmid.2018.02.002. 29486211

[10] Seiffert SN, Carattoli A, Schwendener S, Collaud A, Endimiani A, Perreten V. 2017. Plasmids carrying blaCMY -2/4 in Escherichia coli from poultry, poultry meat, and humans belong to a novel IncK Subgroup Designated IncK2. Front Microbiol 8:407. doi:10.3389/fmicb.2017.00407. 28360894 PMC5350095

[11] Manges AR, Geum HM, Guo A, Edens TJ, Fibke CD, Pitout JDD. 2019. Global extraintestinal pathogenic Escherichia coli (ExPEC) lineages. Clin Microbiol Rev 32:e00135-18. doi:10.1128/CMR.00135-18. 31189557 PMC6589867

[12] Borges CA, Beraldo LG, Maluta RP, Cardozo MV, Barboza KB, Guastalli EAL, Kariyawasam S, DebRoy C, Ávila FA. 2017. Multidrug-resistant pathogenic Escherichia coli isolated from wild birds in a veterinary hospital. Avian Pathol 46:76–83. doi:10.1080/03079457.2016.1209298. 27754714

[13] Wyrsch ER, Nesporova K, Tarabai H, Jamborova I, Bitar I, Literak I, Dolejska M, Djordjevic SP. 2022. Urban wildlife crisis: Australian silver gull is a bystander host to widespread clinical antibiotic resistance. mSystems e0015822. doi:10.1128/msystems.00158-22. 35469421 PMC9238384

[14] Nesporova K, Wyrsch ER, Valcek A, Bitar I, Chaw K, Harris P, Hrabak J, Literak I, Djordjevic SP, Dolejska M. 2020. Escherichia coli sequence type 457 is an emerging extended-spectrum β-lactam resistant lineage with reservoirs in wildlife and food-producing animals. Antimicrob Agents Chemother 65:e01118-20. doi:10.1128/AAC.01118-20. 33020161 PMC7927801

[15] Tarabai H, Wyrsch ER, Bitar I, Dolejska M, Djordjevic SP. 2021. Epidemic HI2 plasmids mobilising the carbapenemase gene blaIMP-4 in Australian clinical samples identified in multiple sublineages of Escherichia coli ST216 colonising silver gulls. Microorganisms 9:567. doi:10.3390/microorganisms9030567. 33801844 PMC7999438

[16] Villa L, García-Fernández A, Fortini D, Carattoli A. 2010. Replicon sequence typing of IncF plasmids carrying virulence and resistance determinants. J Antimicrob Chemother 65:2518–2529. doi:10.1093/jac/dkq347. 20935300

[17] Miriagou V, Papagiannitsis CC, Kotsakis SD, Loli A, Tzelepi E, Legakis NJ, Tzouvelekis LS. 2010. Sequence of pNL194, a 79.3-kilobase IncN plasmid carrying the blaVIM-1 metallo-beta-lactamase gene in Klebsiella pneumoniae. Antimicrob Agents Chemother 54:4497–4502. doi:10.1128/AAC.00665-10. 20660690 PMC2944605

[18] Zelendova M, Papagiannitsis CC, Valcek A, Medvecky M, Bitar I, Hrabak J, Gelbicova T, Barakova A, Kutilova I, Karpiskova R, Dolejska M. 2020. Characterization of the complete nucleotide sequences of mcr-1-encoding plasmids from Enterobacterales isolates in retailed raw meat products from the Czech Republic. Front Microbiol 11:604067. doi:10.3389/fmicb.2020.604067. 33519748 PMC7843963

[19] Jacoby GA. 2009. AmpC beta-lactamases. Clin Microbiol Rev 22:161–182. doi:10.1128/CMR.00036-08. 19136439 PMC2620637

[20] Zhao S, Li C, Hsu C-H, Tyson GH, Strain E, Tate H, Tran T-T, Abbott J, McDermott PF. 2020. Comparative genomic analysis of 450 strains of Salmonella enterica isolated from diseased animals. Genes 11:1025. doi:10.3390/genes11091025. PMC7564550 32883017

[21] Mukerji S, Gunasekera S, Dunlop JN, Stegger M, Jordan D, Laird T, Abraham RJ, Barton M, O’Dea M, Abraham S. 2020. Implications of foraging and interspecies interactions of birds for carriage of Escherichia coli strains resistant to critically important antimicrobials. Appl Environ Microbiol 86:e01610-20. doi:10.1128/AEM.01610-20. 32801178 PMC7531969

[22] Bach S, de Almeida A, Carniel E. 2000. The *Yersinia* high-pathogenicity island is present in different members of the family *Enterobacteriaceae*. FEMS Microbiol Lett 183:289–294. doi:10.1111/j.1574-6968.2000.tb08973.x. 10675599

[23] Vogt D, Overesch G, Endimiani A, Collaud A, Thomann A, Perreten V. 2014. Occurrence and genetic characteristics of third-generation cephalosporin-resistant Escherichia coli in Swiss retail meat. Microb Drug Resist 20:485–494. doi:10.1089/mdr.2013.0210. 24773305

[24] Yamaji R, Friedman CR, Rubin J, Suh J, Thys E, McDermott P, Hung-Fan M, Riley LW. 2018. A population-based surveillance study of shared genotypes of Escherichia coli isolates from retail meat and suspected cases of urinary tract infections. mSphere 3:e00179-18. doi:10.1128/mSphere.00179-18. 30111626 PMC6094058

[25] Poirel L, Decousser JW, Nordmann P. 2003. Insertion sequence ISEcp1B is involved in expression and mobilization of a bla(CTX-M) beta-lactamase gene. Antimicrob Agents Chemother 47:2938–2945. doi:10.1128/AAC.47.9.2938-2945.2003. 12936998 PMC182628

[26] Kurpiel PM, Hanson ND. 2011. Association of IS5 with divergent tandem blaCMY-2 genes in clinical isolates of Escherichia coli. J Antimicrob Chemother 66:1734–1738. doi:10.1093/jac/dkr212. 21636584

[27] Cummins ML, Reid CJ, Djordjevic SP. 2022. F plasmid lineages in Escherichia coli ST95: implications for host range, antibiotic resistance, and zoonoses. mSystems 7:e0121221. doi:10.1128/msystems.01212-21. 35076267 PMC8788324

[28] Chen SL, Hung CS, Xu J, Reigstad CS, Magrini V, Sabo A, Blasiar D, Bieri T, Meyer RR, Ozersky P, Armstrong JR, Fulton RS, Latreille JP, Spieth J, Hooton TM, Mardis ER, Hultgren SJ, Gordon JI. 2006. Identification of genes subject to positive selection in uropathogenic strains of Escherichia coli: a comparative genomics approach. Proc Natl Acad Sci USA 103:5977–5982. doi:10.1073/pnas.0600938103. 16585510 PMC1424661

[29] Cusumano CK, Hung CS, Chen SL, Hultgren SJ. 2010. Virulence plasmid harbored by uropathogenic Escherichia coli functions in acute stages of pathogenesis. Infect Immun 78:1457–1467. doi:10.1128/IAI.01260-09. 20123719 PMC2849428

[30] Huang W-C, Liao Y-J, Hashimoto M, Chen K-F, Chu C, Hsu P-C, Wang S, Teng C-H. 2020. cjrABC-senB hinders survival of extraintestinal pathogenic E. coli in the bloodstream through triggering complement-mediated killing. J Biomed Sci 27:86. doi:10.1186/s12929-020-00677-4. 32762693 PMC7412671

[31] Stephens CM, Adams-Sapper S, Sekhon M, Johnson JR, Riley LW. 2017. Genomic analysis of factors associated with low prevalence of antibiotic resistance in extraintestinal pathogenic Escherichia coli sequence type 95 strains. MSphere 2:e00390-16. doi:10.1128/mSphere.00390-16. PMC5381267 28405633

[32] Elankumaran P, Browning GF, Marenda MS, Reid CJ, Djordjevic SP. 2022. Close genetic linkage between human and companion animal extraintestinal pathogenic Escherichia coli ST127. Current Res Microbial Sci 3:100106. doi:10.1016/j.crmicr.2022.100106. PMC8803956 35128493

[33] Brolund A, Franzen O, Melefors O, Tegmark-Wisell K, Sandegren L. 2013. Plasmidome-analysis of ESBL-producing Escherichia coli using conventional typing and high-throughput sequencing. PLoS One 8:e65793. doi:10.1371/journal.pone.0065793. 23785449 PMC3681856

[34] Tarlton NJ, Moritz C, Adams-Sapper S, Riley LW. 2019. Genotypic analysis of uropathogenic Escherichia coli to understand factors that impact the prevalence of β-lactam-resistant urinary tract infections in a community. J Glob Antimicrob Resist 19:173–180. doi:10.1016/j.jgar.2019.03.002. 30872040 PMC6739196

[35] Dolejska M, Masarikova M, Dobiasova H, Jamborova I, Karpiskova R, Havlicek M, Carlile N, Priddel D, Cizek A, Literak I. 2016. High prevalence of Salmonella and IMP-4-producing Enterobacteriaceae in the silver gull on Five Islands, Australia. J Antimicrob Chemother 71:63–70. doi:10.1093/jac/dkv306. 26472769 PMC4681372

[36] The European Committee on Antimicrobial Susceptibility Testing. Breakpoint tables for interpretation of MICs and zone diameters. Version 9.0, 2019. http://www.eucast.org.

[37] Clinical and Laboratory Standards Institute. 2018. Performance standards for antimicrobial susceptibility testing; 28th informational supplement. CLSI document M100-S28. Clinical and Laboratory Standards Institute, Wayne, PA.

[38] Jouy E, Haenni M, Le Devendec L, Le Roux A, Chatre P, Madec JY, Kempf I. 2017. Improvement in routine detection of colistin resistance in E. coli isolated in veterinary diagnostic laboratories. J Microbiol Methods 132:125–127. doi:10.1016/j.mimet.2016.11.017. 27894831

[39] Bolger AM, Lohse M, Usadel B. 2014. Trimmomatic: a flexible trimmer for Illumina sequence data. Bioinformatics 30:2114–2120. doi:10.1093/bioinformatics/btu170. 24695404 PMC4103590

[40] Bankevich A, Nurk S, Antipov D, Gurevich AA, Dvorkin M, Kulikov AS, Lesin VM, Nikolenko SI, Pham S, Prjibelski AD, Pyshkin AV, Sirotkin AV, Vyahhi N, Tesler G, Alekseyev MA, Pevzner PA. 2012. SPAdes: a new genome assembly algorithm and its applications to single-cell sequencing. J Comput Biol 19:455–477. doi:10.1089/cmb.2012.0021. 22506599 PMC3342519

[41] Walker BJ, Abeel T, Shea T, Priest M, Abouelliel A, Sakthikumar S, Cuomo CA, Zeng Q, Wortman J, Young SK, Earl AM. 2014. Pilon: an integrated tool for comprehensive microbial variant detection and genome assembly improvement. PLoS One 9:e112963. doi:10.1371/journal.pone.0112963. 25409509 PMC4237348

[42] Camacho C, Coulouris G, Avagyan V, Ma N, Papadopoulos J, Bealer K, Madden TL. 2009. BLAST+: architecture and applications. BMC Bioinformatics 10:421. doi:10.1186/1471-2105-10-421. 20003500 PMC2803857

[43] Gurevich A, Saveliev V, Vyahhi N, Tesler G. 2013. QUAST: quality assessment tool for genome assemblies. Bioinformatics 29:1072–1075. doi:10.1093/bioinformatics/btt086. 23422339 PMC3624806

[44] Milne I, Stephen G, Bayer M, Cock PJA, Pritchard L, Cardle L, Shaw PD, Marshall D. 2013. Using Tablet for visual exploration of second-generation sequencing data. Brief Bioinform 14:193–202. doi:10.1093/bib/bbs012. 22445902

[45] Seemann T. 2014. Prokka: rapid prokaryotic genome annotation. Bioinformatics 30:2068–2069. doi:10.1093/bioinformatics/btu153. 24642063

[46] Zankari E, Hasman H, Cosentino S, Vestergaard M, Rasmussen S, Lund O, Aarestrup FM, Larsen MV. 2012. Identification of acquired antimicrobial resistance genes. J Antimicrob Chemother 67:2640–2644. doi:10.1093/jac/dks261. 22782487 PMC3468078

[47] Carattoli A, Zankari E, García-Fernández A, Voldby Larsen M, Lund O, Villa L, Møller Aarestrup F, Hasman H. 2014. In silico detection and typing of plasmids using PlasmidFinder and plasmid multilocus sequence typing. Antimicrob Agents Chemother 58:3895–3903. doi:10.1128/AAC.02412-14. 24777092 PMC4068535

[48] Arndt D, Grant JR, Marcu A, Sajed T, Pon A, Liang Y, Wishart DS. 2016. PHASTER: a better, faster version of the PHAST phage search tool. Nucleic Acids Res 44:W16–21. doi:10.1093/nar/gkw387. 27141966 PMC4987931

[49] Siguier P, Perochon J, Lestrade L, Mahillon J, Chandler M. 2006. ISfinder: the reference centre for bacterial insertion sequences. Nucleic Acids Res 34:D32–6. doi:10.1093/nar/gkj014. 16381877 PMC1347377

[50] Mikheenko A, Valin G, Prjibelski A, Saveliev V, Gurevich A. 2016. Icarus: visualizer for *de novo* assembly evaluation. Bioinformatics 32:3321–3323. doi:10.1093/bioinformatics/btw379. 27378299

[51] Overbeek R, Olson R, Pusch GD, Olsen GJ, Davis JJ, Disz T, Edwards RA, Gerdes S, Parrello B, Shukla M, Vonstein V, Wattam AR, Xia F, Stevens R. 2014. The SEED and the Rapid Annotation of microbial genomes using Subsystems Technology (RAST). Nucleic Acids Res 42:D206–D214. doi:10.1093/nar/gkt1226. 24293654 PMC3965101

[52] Heng L. 2013. Aligning sequence reads, clone sequences and assembly contigs with BWA-MEM. ArXiv 1303.3997. https://arxiv.org/abs/1303.3997.

[53] Langmead B, Salzberg SL. 2012. Fast gapped-read alignment with Bowtie 2. Nat Methods 9:357–359. doi:10.1038/nmeth.1923. 22388286 PMC3322381

[54] Koboldt DC, Zhang Q, Larson DE, Shen D, McLellan MD, Lin L, Miller CA, Mardis ER, Ding L, Wilson RK. 2012. VarScan 2: somatic mutation and copy number alteration discovery in cancer by exome sequencing. Genome Res 22:568–576. doi:10.1101/gr.129684.111. 22300766 PMC3290792

[55] Darriba D, Taboada GL, Doallo R, Posada D. 2012. jModelTest 2: more models, new heuristics and parallel computing. Nat Methods 9:772. doi:10.1038/nmeth.2109. PMC4594756 22847109

[56] Stamatakis A. 2014. RAxML version 8: a tool for phylogenetic analysis and post-analysis of large phylogenies. Bioinformatics 30:1312–1313. doi:10.1093/bioinformatics/btu033. 24451623 PMC3998144

[57] Page AJ, Cummins CA, Hunt M, Wong VK, Reuter S, Holden MTG, Fookes M, Falush D, Keane JA, Parkhill J. 2015. Roary: rapid large-scale prokaryote pan genome analysis. Bioinformatics 31:3691–3693. doi:10.1093/bioinformatics/btv421. 26198102 PMC4817141

[58] Katoh K, Standley DM. 2013. MAFFT multiple sequence alignment software version 7: improvements in performance and usability. Mol Biol Evol 30:772–780. doi:10.1093/molbev/mst010. 23329690 PMC3603318

[59] Price MN, Dehal PS, Arkin AP. 2010. FastTree 2: approximately maximum-likelihood trees for large alignments. PLoS One 5:e9490. doi:10.1371/journal.pone.0009490. 20224823 PMC2835736

[60] Letunic I, Bork P. 2019. Interactive Tree of Life (iTOL) v4: recent updates and new developments. Nucleic Acids Res 47:W256–W259. doi:10.1093/nar/gkz239. 30931475 PMC6602468

