# Supplementary figures and images for: Interspecies Transmission of CMY-2-Producing Escherichia coli Sequence Type 963 Isolates between Humans and Gulls in Australia
Source: mSphere. 2022 Jul 5;7(4):e00238-22. doi: 10.1128/msphere.00238-22 (PMC9429958; doi:10.1128/msphere.00238-22)

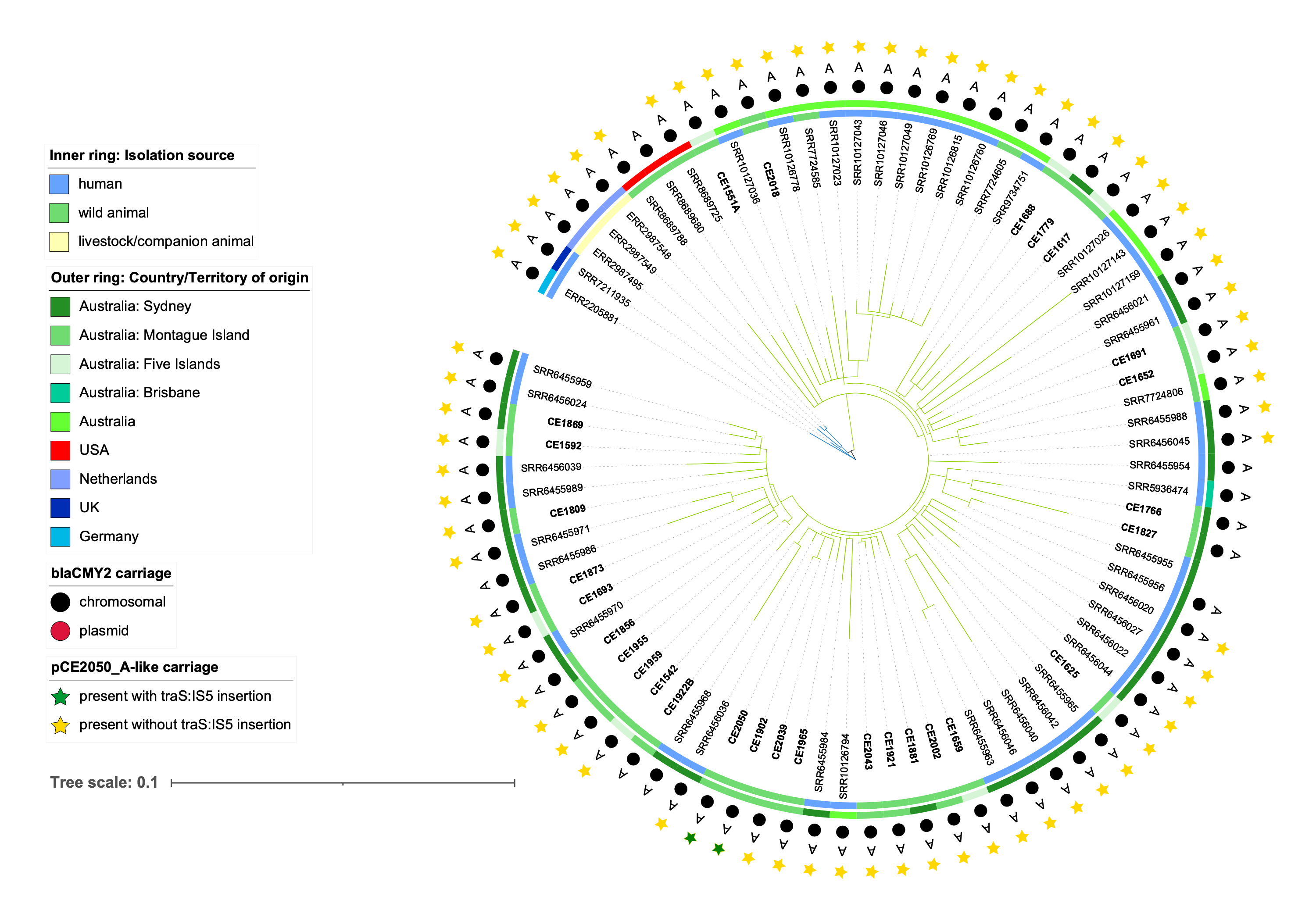

Supplement: FIG S1 [file msphere.00238-22-sf001.tif]
